# Obituary: Dr. Pramod Karan Sethi

**Published:** 2008

**Authors:** Rakesh Bhargava

**Affiliations:** *Professor of Orthopaedics, SMS Medical College, Jaipur, Rajasthan, India.*


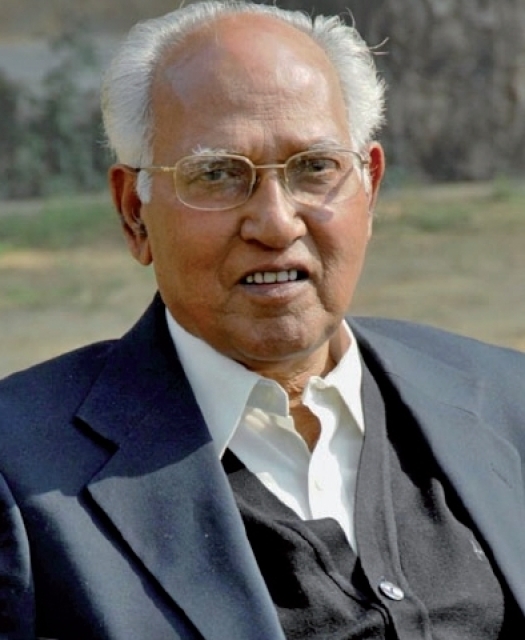


**P. K. Sethi 1927-2008**

Kisse poonche hamne kahan woh chehra-e-roshan dekha hai

Mehfil mehfil ghoom chuke hain, gulshan gulshan dekha hai

Dr. Pramod Karan Sethi was born on 28^th^ November, 1927 in Varanasi. He attended St. John's School (1932-1933), Balwant Rajput Intermediate College (1934-1942), and Agra College (1942-1944). He attained his MBBS (1949) from Sarojini Naidu Medical College, Agra, with honors in Surgery and six other subjects and completed MS in General Surgery (1952). He obtained FRCS in 1954. On his return to India, Dr. Sethi joined as a lecturer in Surgery at the SMS Medical College and Hospital, Jaipur, with which he remained associated till his retirement in 1982. He started and developed Orthopaedic Department and Rehabilitation Unit from scratch.

Dr. Sethi sometimes met his amputees whom he had fitted with prosthesis, but who were not wearing it. He soon realized the reason was the foot piece, which did not fit their milieu. He set out to solve the rural patients' need for a foot piece that would look like a bare foot, would be waterproof and durable and flexible enough to allow for ease of walking over uneven ground and for its wearer to squat and sit cross-legged. Finally, it should be made of inexpensive, readily available materials. Thus, began the journey of the development of the Jaipur foot. Ram Chandra, a master craftsman whom Dr. Sethi affectionately referred to as “Masterji,” produced an aluminum die. Dr. Sethi, drawing on the experience in Sri Lanka of surgeon G. M. Muller, decided to pack the die with rubber. Knowing “next to nothing about rubber,” he asked Chuga Bhai, owner of a one-man tire retreading shop near the hospital to vulcanize the foot, but it was too heavy. The doctor and the craftsman produced modification after modification. Finally, the SACH foot assembly was eliminated completely and different components were used. The final product had flexibility and resilience, simulated subtalar, ankle, and torsional movement and it was durable. Dr. Sethi's desire and hope was that the Jaipur model of uniting the skills and services of doctors, craftsmen, and volunteers be duplicated throughout India so that the handicapped can be served closer to their homes.

He presented his first scholarly paper on the Jaipur foot at the Association of Surgeons of India's annual conference, Bangalore (1970). That same year, he reported on the foot at a meeting of the British Orthopedic Association at Oxford, England and received a standing ovation. The Western India Orthopedic Society presented Dr. Sethi with a Gold Medal in 1973. Dr. Sethi was asked to give the lead talk at the First World Congress on Prosthetics and Orthotics held in Montreux, Switzerland (1974). He was conferred Padma Shri in 1981, Ramon Magsaysay Award, Manila (1981), Guinness Award for Scientific Achievement (1982), D.Sc. (Honoris Causa), Rajasthan University (1982), and R.D. Birla Award for Outstanding Medical Research (1983). He delivered the Gandhi Memorial Oration (1988), Raman Research Institute, Bangalore and was elected a Fellow of the Indian Academy of Sciences (1989). He was awarded the Knud Jansen Medal and Oration, World Congress in Prosthetics and Orthotics, Kobe, Japan (1989). He received the Dr. B.C. Roy National Award as Eminent Medical Man (1989), and an Honorary Fellowship, Indian Orthopaedic Association (1999).

His research endeavors were not limited to the Jaipur foot alone. Dr. Sethi and Prof. S.C. Lakkad of IIT Mumbai worked on using carbon fiber composites for fabricating light-weight calipers, a spin-off of aerospace, and defense technology for social welfare initiated by the former President A. P. J. Abdul Kalam, then Director of Defense Research and Development Organization (DRDO). This was standardized by DRDO and Nizam Institute of Medical Sciences, Hyderabad, in 1994. Till now 9000 polio patients have benefited all over the country with this technology.

His excellent professional knowledge was indisputable. He spent at least 1 or 2 h every single day in the Robert Heilig library of SMS Medical College. He was an avid reader. He was a Member of an elite book lovers club, which included luminaries of the Rajasthan University as Dr. Daya Krishan, a professor of Philosophy; Prof. Unnithan, a musicologist; Dr. Mukund Laat, a physicist; Prof. Loknathan, Dr. Rao of Malviya Engineering College, who was also a scholar of nature study; Ms. Hemlata Prabhu, a professor of English, and Mr. Anil and Mrs. Otima Bordia. This diverse group met once a month to review a book, be it the latest best seller, fiction or nonfiction, on literature or arts, metaphysics or philosophy, on management or behavior, and so on. No wonder that his personal library is probably among the best individual collection of books.

The first qualities of the heart a surgeon must possess and which he did in ample measure was humility. It is reflected in his response to the citation for the R.D. Birla National Award, which seemed to come straight from the heart. “The RD Birla Smarak Kosh has honoured our small team of doctors and artisans and the community of my town of Jaipur without whose help and contribution it would not have been possible. For years our work went unnoticed because there was nothing exotic or glamorous about the simple, almost austere technology which was associated with it….the present award has lent credibility and legitimacy to the kind of clinical research which is meaningful and relevant for our country's needs. Hopefully our research institutions and planning bodies would now be compelled to carry out some hard reappraisal of their priorities.”

In qualities of the hand, he was a master craftsman. At surgery, he invariably meticulously planned his case. He used to tell us that, no matter how many times he had performed an operation, he will review it before coming for surgery. His dissection was meticulous, patiently executed, and no step skipped. He would set out problems and tasks and indicate a line along which they may be approached.

Dr. Sethi had a very versatile personality, coupled with a charming and pleasant countenance. Many of his postgraduates joined Orthopaedics not out of a genuine love or craze for the subject, but because of the charisma of this man, Dr. P. K. Sethi. I was one among them. In the words of William Shakespeare “Here was a Caesar. When comes such another.”

Dr. P. K. Sethi passed away in the early hours of 6^th^ January, 2008. He is survived by his wife, Sulochna; three daughters, Lata, Nita and Amrita; and a son, Harsh.

